# Neural Activity during Natural Viewing of *Sesame Street* Statistically Predicts Test Scores in Early Childhood

**DOI:** 10.1371/journal.pbio.1001462

**Published:** 2013-01-03

**Authors:** Jessica F. Cantlon, Rosa Li

**Affiliations:** Department of Brain and Cognitive Sciences, Rochester Center for Brain Imaging, University of Rochester, New York, New York, United States of America; University of Oregon, United States of America

## Abstract

It is not currently possible to measure the real-world thought process that a child has while observing an actual school lesson. However, if it could be done, children's neural processes would presumably be predictive of what they know. Such neural measures would shed new light on children's real-world thought. Toward that goal, this study examines neural processes that are evoked naturalistically, during educational television viewing. Children and adults all watched the same *Sesame Street* video during functional magnetic resonance imaging (fMRI). Whole-brain intersubject correlations between the neural timeseries from each child and a group of adults were used to derive maps of “neural maturity” for children. Neural maturity in the intraparietal sulcus (IPS), a region with a known role in basic numerical cognition, predicted children's formal mathematics abilities. In contrast, neural maturity in Broca's area correlated with children's verbal abilities, consistent with prior language research. Our data show that children's neural responses while watching complex real-world stimuli predict their cognitive abilities in a content-specific manner. This more ecologically natural paradigm, combined with the novel measure of “neural maturity,” provides a new method for studying real-world mathematics development in the brain.

## Abstract 2

Neural activity that is evoked naturalistically in children during educational television viewing can be used to predict math and verbal knowledge.

## Author Summary

In the real world, children learn new information by participating in classrooms, interacting with their family and friends, and watching educational videos. While previous neuroimaging research has typically used simple tasks and short-lasting stimuli, in this study we examined brain development using a more complex and naturalistic educational stimulus. Children and adults all watched the same *Sesame Street* video as we measured their neural activity using functional magnetic resonance imaging (fMRI). We examined the timecourses of neural activity over the length of the video for children and adults. We found that the degree to which children showed adult-like brain responses was correlated with their math and verbal knowledge levels. In the intraparietal sulcus, children's neural correlation with adults depended on their mathematics knowledge whereas in Broca's area, it depended on their verbal knowledge. Additional experiments showed that children's neural responses in the intraparietal sulcus are selectively driven by numerical content both when children are watching *Sesame Street* and when they engage in a number matching task. These convergent results highlight the broad role of the intraparietal sulcus in processing numerical information. In addition, our study validates the use of naturalistic stimuli and child-to-adult neural timecourse correlations for studying brain development. We suggest that this new approach can enrich our understanding of how children's brains process information in the real world.

## Introduction

Naturalistic thought is an important phenomenon to understand in children who spend most of their time absorbing new information from complex scenes such as homes, schools, computers, and televisions. There is recent interest in neural activity that occurs spontaneously when people watch a natural scene or movie [1]–[3]. Naturalistic neuroimaging studies open up opportunities to collect neural measurements of children's unconstrained thoughts during real-world stimulus viewing. In this study we ask whether children's neural activity during unconstrained natural viewing of educational videos statistically predicts their performance on mathematics and verbal tests.

Advances in developmental functional magnetic resonance imaging (fMRI) have been rapid considering that the practice of scanning children in fMRI studies began less than 20 y ago [4]. Traditional fMRI studies of category and concept development often test neural processes under conditions of maximal stimulus control (e.g., isolated pictures, tones, words, letters, or digits) with short-duration stimuli and equally short response times (i.e., 2 s). These types of studies are critical for understanding brain development, and considerable progress has been made toward understanding all aspects of brain development using a diverse array of controlled tasks in children; see [5]–[7] for review. However, the general approach of using stripped down experimental designs could present a limitation on a broad understanding of child development, as the types of thoughts that a child has in a 2-s time window with uncomplicated tasks and stimuli may not be as diagnostic of their cognitive development as how they think over long periods of time with more complex stimulation. The more traditional neuroimaging approach of using highly controlled, simple stimuli and tasks could be complemented by an approach that tests children's neural responses under more complex real-world conditions.

As a first step toward interpreting children's real-world neural activity, we tested the relationship between children's natural viewing neural activity and their school-based knowledge. We focused on mathematics development because substantial progress has been made in characterizing the neural profile of calculation in adults [8],[9], children [10]–[12], and non-human primates [13]. The data consistently indicate that regions of intraparietal cortex are more responsive during numerical processing compared to processing of other stimulus classes such as colors [14], shapes [15],[16], faces, and words [16],[17], as well as actions such as grasping and saccadic eye movements [17]. Moreover disruption of normal functioning in intraparietal cortex through cortical lesions [18] and genetic disorders [19] is associated with selective impairments for numerical processing. Here we ask whether children show number-specific neural responses during a typical early childhood educational experience by testing the maturity of children's neural timecourses as they view educational videos. In addition, we test for a dissociation between the neural correlates of children's school-based mathematics and verbal test scores in order to examine whether there are dissociable, content-specific patterns in children's brain activity during natural viewing. Finally, we compare the neural measure derived from natural viewing with a neural measure from a traditional fMRI task to determine the relative strengths of those measures as statistical predictors of children's math performance.

## Results

**Figure 1:**
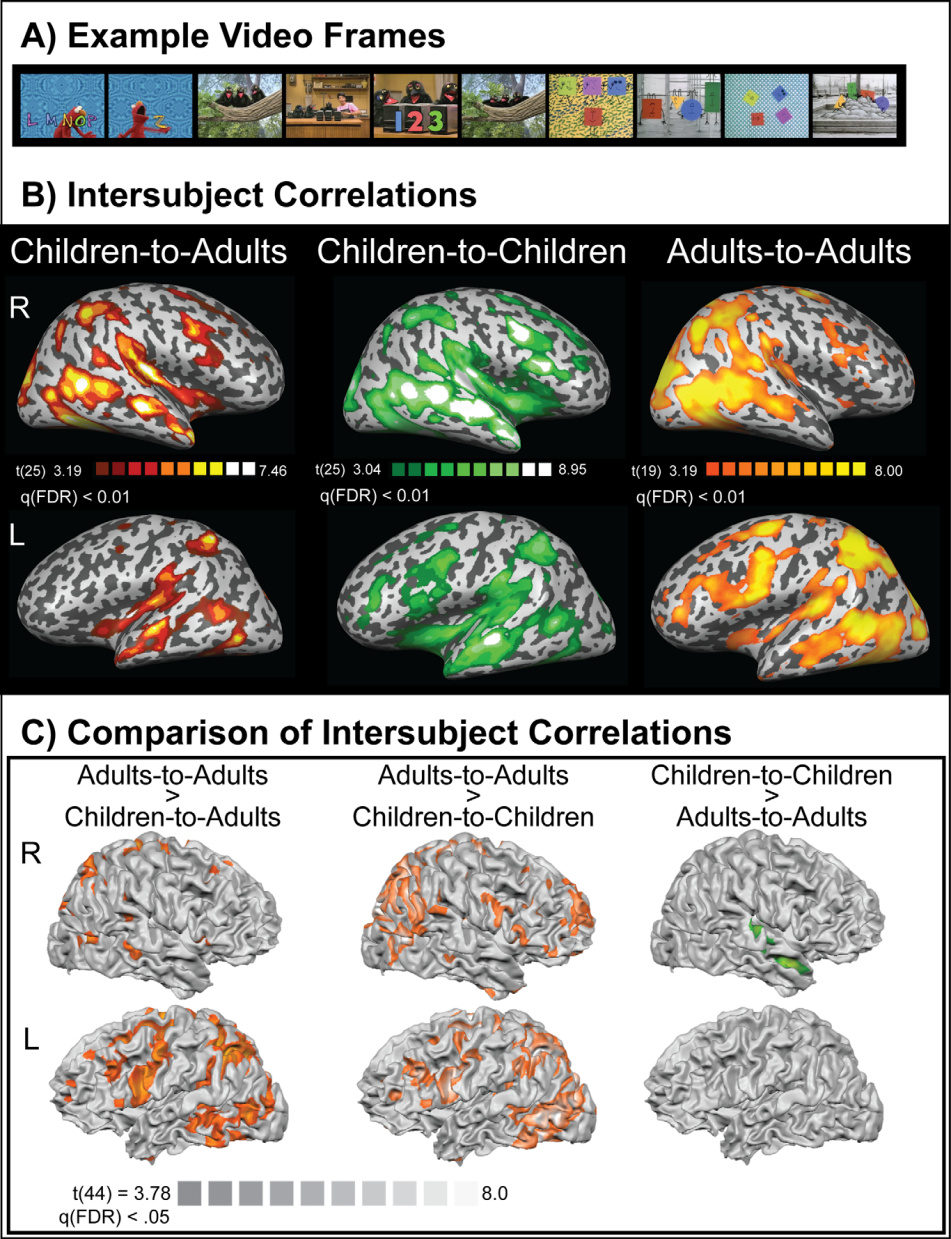
Example stimuli and intersubject correlations. (A) Example frames from the educational television series in the natural viewing movie paradigm. (B) The average group-level intersubject correlation in BOLD time-series during the 20-min movie (Fisher transformed one-sample *t*-test over individual children's mean r-maps versus the group mean correlation value; FDR-corrected, *q*<0.01) for children correlated with adults (left), children correlated with children (middle), and adults correlated with adults (right). (C) Whole-brain statistical comparisons over intersubject correlations for children-to-adults, adults-to-adults, and children-to-children (Fisher transformed unpaired *t*-tests over individual subject mean r-maps).

As described in Materials and Methods, we used an intersubject correlation method [2] to measure the similarity of children's neural responses to those of adults after both groups watched the same 20-min *Sesame Street* video (Figure 1A). Our version of the intersubject method correlated the whole neural timecourse at every voxel in the brain between each child and a group of adults. From this correlation we derived a measure for each child of how “adult-like” or, mature, his/her pattern of neural activation was at each voxel. These maps are designated “neural maturity” maps. We performed group statistics (Fisher-transformed one sample *t*-tests) over the children's “neural maturity” maps (Figure 1B). The map shows regions where the similarity in neural timecourses between the children and adults while watching the video was consistently high at the group level. Broadly speaking, the children showed group-level similarity to adults in cortical regions associated with vision (occipital cortex), auditory processing (lateral temporal cortex), language (frontal and temporal cortex), visuo-spatial processing and calculation (intraparietal cortex), and several other functions. For comparison, Figure 1B also shows the mean intersubject correlation within the group of children (middle panel) and within the group of adults (right panel). The intersubject correlations among subjects reinforce claims that there are certain universals in the way that the human brain processes information [2]. We found that the intersubject correlations of children-to-adults, which we have termed “neural maturity,” increased with age. Neural maturity increased with age across large sections of the brain including basic sensory and motor cortices as well as areas of association cortex such as the intraparietal sulcus (IPS) and Broca's area (Table 1).

**Table 1:** Regions showing correlations between “neural maturity” and the factors of age, verbal score, and math score in children.

Correlations	Peak X	Peak Y	Peak Z	Region	Peak r
**Age**	−52	7	18	Broca's area/BA 44	0.73
	−43	−59	0	BA 37	0.76
	−43	31	6	BA 46/47	0.79
	−40	31	7	BA 46	0.76
	−34	−8	42	BA 6	0.72
	−31	−41	42	IPS, BA 40/7	0.83
	−22	1	−3	Striatum	0.84
	−7	22	39	Cingulate	0.68
	2	−20	−9	Midbrain	0.79
	5	−51	18	Cingulate	0.84
	23	−5	9	Striatum	0.73
	26	−62	−6	BA 19	0.81
	32	−62	18	BA 19	0.79
	47	−32	36	IPS, BA 40/7	0.82
	47	−17	30	BA 2	0.81
	47	7	18	BA 44	0.80
**Verbal**	−52	13	15	Broca's Area,/BA 44	0.69
	−22	−17	27	Caudate	0.69
	−19	−71	−25	Cerebellum	0.71
	11	−92	−18	Cerebellum	0.72
	20	−23	12	Thalamus	0.58
	26	−50	−18	BA 37	0.71
	26	−5	24	Caudate	0.80
	33	−11	24	Insula	0.67
	35	−80	−24	Cerebellum	0.72
	41	−14	57	BA 4	0.76
**Math**	−31	−89	21	BA 19	0.84
	−22	−62	45	IPS, BA 40/7	0.79
	11	55	48	Precuneus	0.69
	32	−56	57	IPS, BA 40/7	0.64
	53	13	39	BA 8	0.79

BA, Brodmann's area.

A whole-brain analysis comparing the intersubject correlations of children-to-adults with adults-to-adults revealed statistically higher intersubject correlations in adults-to-adults than children-to-adults predominantly in left hemisphere cortex including the left IPS, left Broca's area and the inferior and middle frontal gyri, left superior temporal sulcus, and the left fusiform and inferior temporal gyri (Figure 1C). These results complement the age-related increases in neural maturity that we report in Table 1 and indicate that neural responses that are universal among adults are still maturing in children, particularly in the left hemisphere. Interestingly, the statistical comparison of intersubject correlations for children-to-children versus children-to-adults indicated that children exhibit statistically higher intersubject correlations with other children than with adults in superior temporal cortex, predominantly in Brodmann area 22. This is an interesting finding because it suggests that there are brain regions in which children's neural responses are still immature but the pattern of neural responses is systematic among children. We also tested a whole-brain analysis comparing children-to-children intersubject correlations with adult-to-adult intersubject correlations. Children showed significantly higher intersubject correlations than did adults in superior temporal cortex along Brodmann 22. We note that the higher intersubject correlations among children in superior temporal cortex were bilateral at a slightly lower threshold than shown in Figure 1C. The fact that children show significantly higher intersubject correlations than adults in this region indicates that it not only exhibits a higher correlation among children than between children and adults but it also exhibits less between-subject variability in neural activity in childhood than in adulthood. Maps of the statistical differences in intersubject correlations between children and adults are presented in Figure 1C.

**Figure 2:**
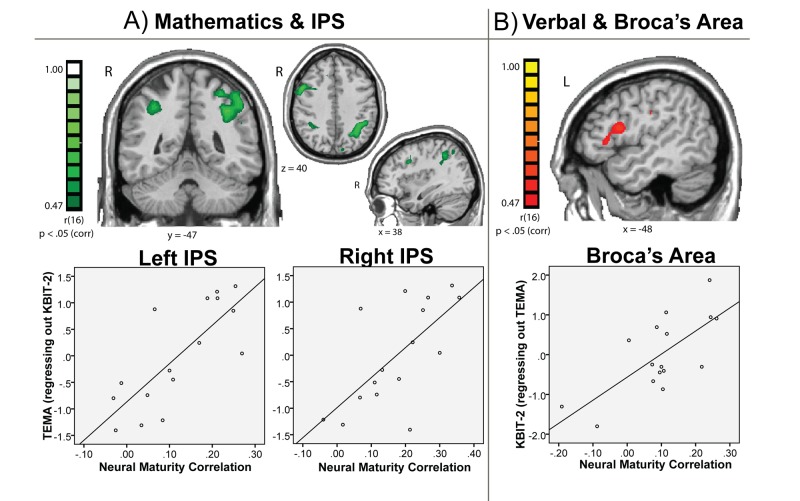
Whole-brain correlation of natural viewing neural maturity and test scores. (A) Children's intersubject correlation to adults in the IPS bilaterally predicts their performance on a mathematics IQ test (TEMA), independently of their KBIT IQ test performance (whole brain analysis, cluster corrected at *p*<0.05, minimum 88 voxels). The figure shows regions that emerged from the whole brain correlation (top) and the mean correlation across the left and right IPS clusters (bottom). (B) Children's neural maturity in the left inferior frontal gyrus (Broca's area) predicts their performance on the KBIT verbal IQ test after regressing out their mathematics performance (TEMA). The top panel shows the results of the whole brain analysis (whole brain analysis, cluster corrected at *p*<0.05, minimum 65 voxels). The bottom panel shows the mean correlation across the Broca's area cluster to illustrate individual subject data.

We next explored the relation between the child-to-adult intersubject correlations, which we are calling “neural maturity,” and behavior. Children were administered the TEMA-3 and KBIT-2, standardized tests for childhood mathematics and verbal/non-verbal IQ, respectively. In a voxelwise whole brain analysis, test scores were correlated with children's neural maturity values. This analysis returned a map of correlation coefficients relating individual variability in test scores with individual variability in neural maturity for the whole brain (Figure 2). These brain-behavior correlation maps represent the correlation of one standardized test while controlling for the other standardized test score in a partial correlation.

**Figure 3:**
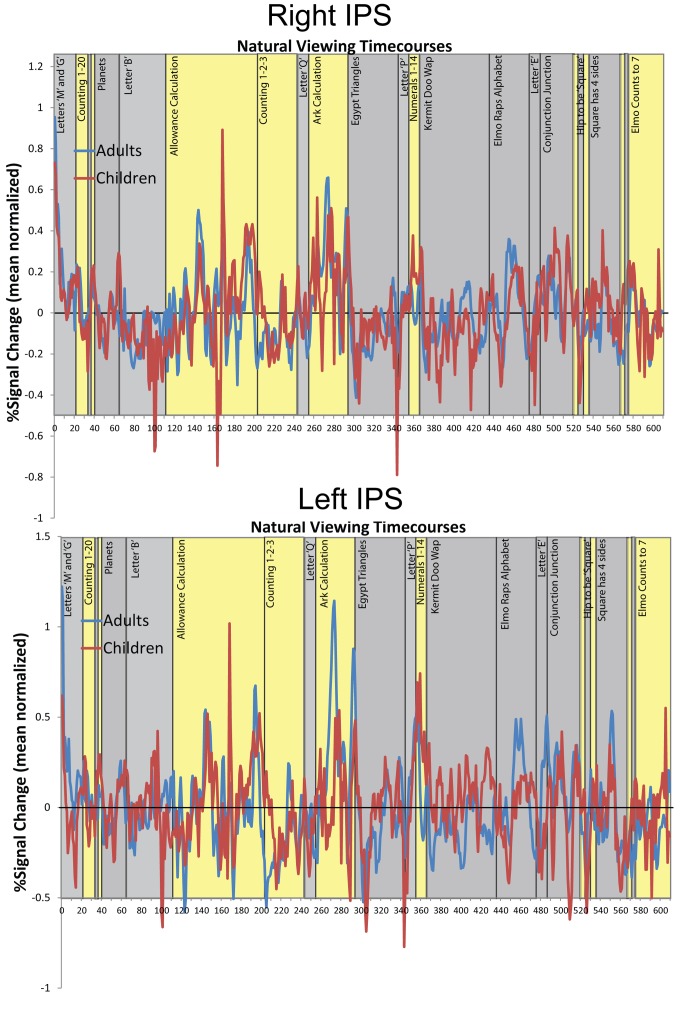
BOLD timecourses of activation from the IPS regions from adults (blue) and children (red) from the video series. The timecourses are averaged across the IPS voxels from Figure 2A. Percent signal change is calculated relative to the mean amplitude of the timecourse (the zero ordinate represents mean amplitude). The yellow bars indicate time periods where numerical information was presented in the video and the gray bars represent non-numerical video segments. The timecourses illustrate the correlation between the neural responses of children and adults.

Figure 2A shows the resulting maps of the whole brain analysis of children's neural maturity correlated with their performance on the TEMA-3 mathematics test (controlling for their KBIT-2 scores). The data show that in bilateral regions of the IPS, children's neural maturity predicted their performance on the standardized math test, independently of how they performed on the KBIT-2 test. That is, children who performed better specifically on the math test exhibited more similar IPS responses to adults while watching the educational videos. The bottom panel of Figure 2A illustrates the average correlation between children's neural maturity values and math test scores for the left and right IPS regions of interest (ROIs). The ROI-averaged neural maturity values were calculated by taking each subject's average timecourse for the whole ROI and correlating that timecourse with the group average timecourse from the adults. The group-level correlation between the natural viewing neural timecourses of children and adults can be seen in Figure 3, which shows the raw ROI-averaged timecourses for the left and right IPS, for each group. The timecourses show comparable patterns of peak responses for children and adults across the video series in the IPS. The residual timecourses after framewise displacement (FD) correction are shown in Figure S1 for both subject groups along with the average child-to-adult correlation for each brain region.

We tested whether our brain-behavior correlation between math test scores and IPS neural maturity could be explained by some other variable related to the scanning session. We found that the correlation between math test scores and IPS neural maturity is not explained by general memory and attention because neural maturity in those same IPS voxels did not correlate with children's scores on a general memory test about the video (left: R = −0.07, *p* = 0.41; right: R = 0.32, *p* = 0.12). In addition, the relationship between neural maturity and math test scores was not attributable to individual differences in head motion as the correlation remained significant when motion (translation and rotation) and KBIT-2 scores were simultaneously controlled (right: R = 0.67, *p*<0.01; left: R = 0.76, *p*<0.01).

**Figure 4:**
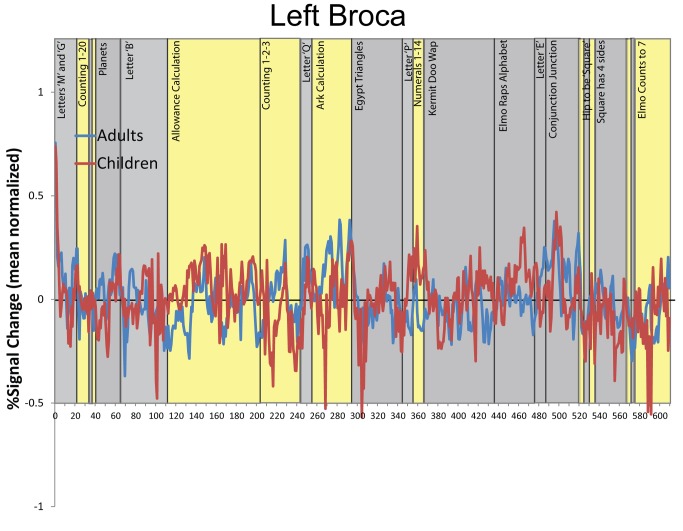
BOLD timecourses of activation from Broca's area from adults (blue) and children (red) from the video series. The timecourses are averaged across the Broca's area voxels from Figure 2B. Percent signal change is calculated relative to the mean amplitude of the timecourse (the zero ordinate represents mean amplitude). The yellow bars indicate time periods where numerical information was presented in the video and the gray bars represent non-numerical video segments.

We performed a parallel analysis with the standardized verbal IQ test scores (KBIT-2 verbal) to test for a functional dissociation between the mathematics and verbal domains. Figure 2B shows regions where children's neural maturity was correlated with their performance on the KBIT-2 verbal test, controlling for TEMA-3 performance. This analysis yielded a different pattern of regions including Broca's area and ventral temporal cortex. Figure 2B (bottom panel) illustrates the relationship between neural maturity and verbal test scores in Broca's area calculated from individual subjects' ROI-averaged timecourses. The correlation between verbal test scores and neural maturity in Broca's area remained significant when math test scores and motion parameters were simultaneously controlled (R = 0.68, *p*<0.01). Figure 4 shows the ROI-averaged timecourses for children and adults in Broca's area across the natural viewing movie sequence. Broca's area has been previously reported to respond during picture naming and verb generation tasks, consistent with our finding that it relates to children's formal verbal abilities [20]–[23]. However, the main finding is that the relationship between natural viewing neural maturity and math test scores is dissociable from the relationship between neural maturity and verbal test scores, implicating content-specific processing during natural viewing. Table 1 reports all of the brain regions that exhibited content-specific correlations between neural maturity during natural viewing and the math and verbal test scores in the whole brain analyses.

**Figure 5:**
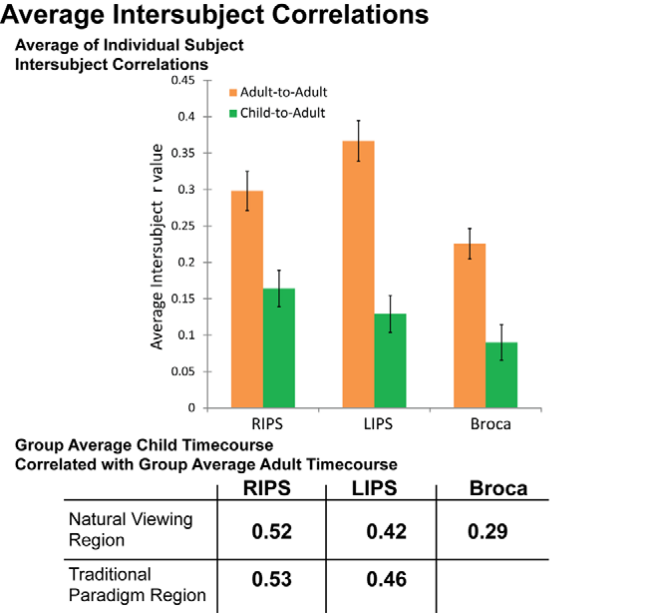
Average intersubject correlation for adults-to-adults and children-to-adults in each of the three ROIs from **Figure 2**: right IPS, left IPS, and Broca's area. In the top panel, intersubject correlations were calculated between each subject and the group mean (never including a subject's own data in the group mean for adult-to-adult correlations). In the bottom panel, the chart presents the correlation values when the correlation is calculated over the group mean timecourses for each region. The correlations were calculated across the 609 timepoints shown in Figures 3 and 4.

Figure 5 shows a summary of the mean natural viewing intersubject correlation values for children-to-adults (neural maturity) and adults-to-adults in the left and right IPS and Broca's area. Adults showed a significantly higher intersubject correlation with other adults than did children with adults in all three regions, providing further evidence that the intersubject correlation during natural viewing strengthens over development (all *p*-values<0.001).

**Figure 6:**
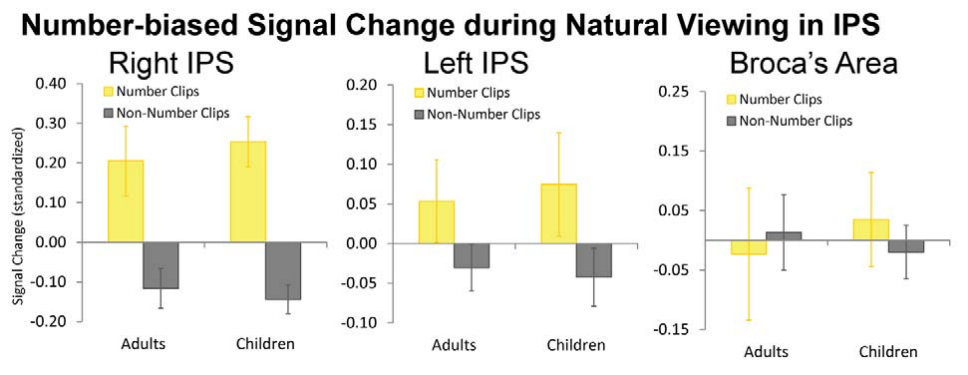
Percent signal change for the numerical content versus the non-numerical content of the natural viewing video in adults and children. Percent signal change in the right IPS is significantly higher for the numerical content than the non-numerical content. The left IPS shows a marginally non-significant trend toward higher activation during numerical content than non-numerical content. Broca's area shows no difference between the numerical and non-numerical content.

In order to test whether the neural responses in the IPS during the natural viewing session were driven primarily by the numerical content portions of the *Sesame Street* video, we analyzed changes in response amplitude over the timecourses relative to the content of the movie. As mentioned earlier, Figures 3 and 4 show changes in percent signal change over the course of the movie as well as the timing of the movie content. The baseline (the zero ordinate) in Figures 3 and 4 is the mean timecourse value. We found that the right IPS region exhibited a significantly greater response amplitude during the numerical content than the non-numerical content from the video (Figure 6; *t*(22) = 3.58, *p*<0.01). The left IPS exhibited greater responses to numerical content compared to non-numerical content, but the difference was not significant. Broca's area did not exhibit a significant difference in percent signal change between numerical and non-numerical clips. Figure 6 shows the response amplitude differences between numerical and non-numerical clips for the right IPS, left IPS, and Broca's area. Importantly, we observed that the IPS intersubject correlations were not driven exclusively by these differences in amplitude between the numerical and non-numerical content because the intersubject neural maturity correlation was significant during both the numerical content and non-numerical content in both IPS regions (Fisher transformed r versus zero; left: numerical *t*(22) = 3.14, *p*<0.005, non-numerical *t*(22) = 4.40, *p*<0.001; Right: numerical *t*(22) = 5.23, *p*<0.001, non-numerical *t*(22) = 4.97, *p*<0.001). This shows that blood oxygen level dependent (BOLD) amplitude and intersubject correlation are distinct measures of brain development because neural responses are systematic and temporally correlated between subjects even during the presentation of stimuli for which the IPS does not show a selective, high-amplitude BOLD response. The implication is that there is a systematic temporal pattern even in the low-amplitude BOLD responses.

**Figure 7:**
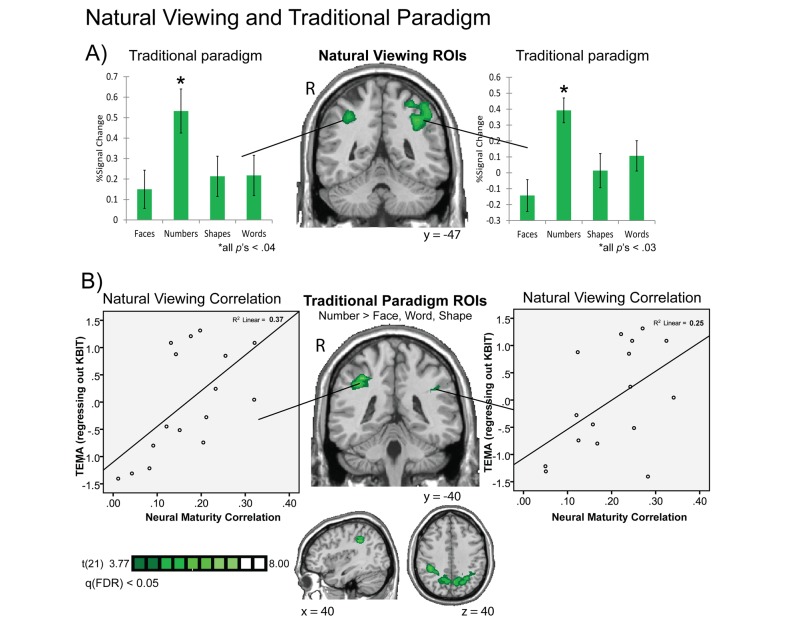
Cross-comparison of natural viewing and traditional task results. (A) IPS regions that exhibited a correlation between children's natural viewing neural maturity and math IQ test score also exhibited a selective response to numerical stimuli compared to face, shape, and word stimuli in a traditional fMRI paradigm. (B) And vice versa, IPS regions that exhibited a significantly greater response to the numerical stimuli in a traditional fMRI paradigm in children (numbers > faces + words + shapes, FDR corrected, *p*<0.05) also exhibited a significant correlation between children's neural maturity from the natural viewing paradigm and their mathematics test scores (TEMA-3).

In a second experiment, we tested the same children in a more traditional fMRI paradigm to validate our natural-viewing method. In this traditional paradigm, the children were tested on a matching task with isolated pairs of faces, numbers, words, and shapes. We tested whether the ROIs that emerged from the neural maturity correlations also elicit content-specific responses during a more controlled, traditional fMRI paradigm. Figure 7A shows the children's neural response amplitudes for each of the four stimulus classes from the traditional paradigm inside the IPS ROIs that were defined as showing a relationship between children's neural maturity and math test scores during natural viewing. The data from this more traditional fMRI paradigm indicate that the IPS responded more strongly to numerical stimuli than to the three classes of non-numerical stimuli during the traditional matching task. This result accords with our finding that the maturity of children's neural responses in the IPS during educational video viewing has a biased relation to mathematics processing and is not generically related to intelligence.

As described earlier, the bias for numerical processing in the IPS has been well established by several previous traditional fMRI studies with adults as well as children [8],[10],[11],[14]; see [9],[12] for review. So far no study has demonstrated a relationship between young children's neural amplitudes during traditional fMRI tests of numerical processing and their formal school-based math test performance. We tested whether children's number-related BOLD amplitudes from the traditional paradigm would predict their math test scores. We did not find a correlation between number-related amplitudes and children's math test performance in the traditional paradigm (left IPS: R = −0.17, *p* = 0.53; right IPS: R = −0.29, *p* = 0.25). This result contrasts with our findings from the natural viewing neural measures that showed a significant correlation between children's IPS activity and math performance. One explanation of the difference in results is that the content of the math-related material in the educational video is more closely related to children's school-based math skills, which results in a better correlation between the natural viewing neural data and the math test scores. This raises the possibility that neural responses to real-world stimuli might be better predictors of full-blown math development than neural responses from a simpler traditional fMRI task.

**Figure 8:**
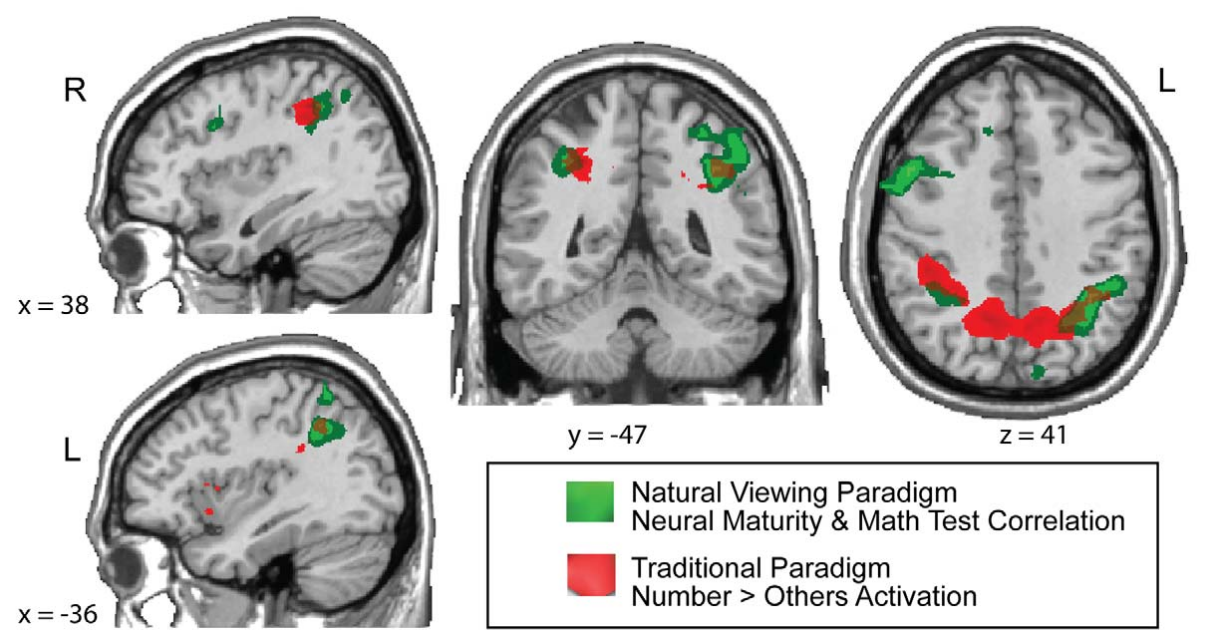
Illustration of overlap in IPS activation between the math-related activation from the natural viewing task (from **Figure 2A**) and the number-related activation from the traditional task (from **Figure 7B**). The statistical thresholds and parameter ranges are the same as in the original figures: Figures 2A and 7B.

As a cross-validation of our results, we tested whether our natural viewing intersubject correlation results are maintained when the IPS is defined by activation during the traditional numerical tests. We selected the clusters of parietal voxels that elicited a statistically greater response during the traditional number task than the face, shape, and word-matching tasks (whole-brain, random effects analysis; *n* = 22 children; number matching > face, word, and shape matching; false discovery rate [FDR] corrected, *q*<0.05). These intraparietal ROIs, now defined by activation during a traditional fMRI task, showed the same partial correlation between children's math IQ scores and their neural maturity correlations during the *Sesame Street* video, controlling for KBIT scores (Figure 7B). Moreover, the spatial distribution of children's neural responses to numbers from the traditional task overlapped with the brain regions that showed a correlation between children's natural viewing neural maturity measures and their formal math test scores. Figure 8 shows the spatial overlap of the natural viewing and traditional task results in the IPS (whole-brain results are plotted for both datasets in Figure 8). The IPS overlap between these two maps is impressive given that one result (natural viewing neural maturity) represents the relation between children's math test scores and their child-to-adult timecourse correlations from watching *Sesame Street* while the other result (traditional task) represents children's neural responses to numbers over other stimulus categories from a matching task.

Finally, we used the traditional task number-related ROIs as an independent localizer to test the relationship between math test scores and the natural viewing neural responses during the numerical versus non-numerical content of the video. We tested the correlation between children's math test scores and (1) natural viewing neural maturity for the numerical versus non-numerical video content, and (2) response amplitude for the numerical versus non-numerical video content. We controlled for KBIT-2 test scores and motion in these analyses. We found that the right IPS, defined by the traditional numerical task, showed a significant correlation between math test scores and the neural maturity natural viewing correlation only for the numerical video content (one-tailed tests; numerical: R = 0.52, *p*<0.05; non-numerical: R = 0.37, *p* = 0.11). In addition, response amplitude during only the numerical content of the video was significantly correlated with children's math test scores (R = 0.63, *p*<0.05). Response amplitude to the non-numerical video content was negatively correlated with math test scores because of its negative correlation with response amplitude to numerical content (R = −0.63). The left IPS showed less of a distinction between numerical and non-numerical content in the correlation with math test scores as correlations for both content types were significant or marginally significant (numerical: R = 0.39, *p* = 0.09; non-numerical: R = 0.55, *p*<0.05). Response amplitude for numerical video content was also marginally correlated with math test scores in the left IPS (R = 0.37, *p* = 0.10).

Reviewing the neural measures from both the natural viewing and traditional paradigm, we found that the right IPS appears to be more mature than the left IPS in children. The right IPS showed a higher neural maturity score than left IPS in regions defined both by the traditional task and the natural viewing task (Fisher transformed paired *t*-tests; traditional: *t*(22) = 2.41, *p*<0.05; natural: *t*(22) = 2.74, *p*<0.01). The general pattern is that the temporal response pattern in the right IPS in children shows more similarity to the adult IPS than does the left IPS. Although both regions showed strong correlations between neural maturity and mathematics performance, the right IPS correlation between neural maturity and math test scores was specific to the numerical content of the video while the left IPS response was not. Compared to the left IPS, the right IPS is considered to play a greater role in the early stages of numerical development [12]. Our natural viewing data suggest that both the left and right IPS are important for mathematics development in early childhood but that the right IPS matures faster than the left IPS and its response is more selectively modulated by numerical content in early childhood.

In summary, the results from the natural viewing paradigm demonstrate that the whole timecourse of neural activation from fMRI (not just the neural amplitude) carries important information about cognitive and brain development. The data indicate that in early childhood the IPS (particularly in the right hemisphere) responds in a content-specific manner to numerical information presented naturalistically and that both the amplitude and temporal pattern of the neural response are related to children's school-based math performance. A comparison of the natural viewing and traditional paradigms shows that number-selective responses from the two paradigms overlap in parietal cortex. Both paradigms indicate content-specificity in the IPS for numerical processing in children. Yet, only the neural measures from the natural viewing paradigm correlated with children's formal school-based math performance. This suggests that some aspect of the stimulus content or measurement from the natural viewing paradigm is better able to represent the neural basis of children's early math performance than the traditional paradigm.

## Discussion

We used a novel fMRI method to show that naturalistic neural activity is related to school-based mathematics knowledge in children. Specifically, the similarity in children's IPS neural timecourse to that of adults during natural viewing predicts their mathematics test performance. The relationship between naturalistic IPS activity and math performance is dissociable from the relationship between natural viewing activity in Broca's area and children's verbal IQ performance. In addition to showing a mathematics-related temporal pattern in the IPS, children's IPS responses during the numerical segments of the *Sesame Street* video were also higher in amplitude than during the non-numerical video segments. Together these findings demonstrate content-based neural responses during natural viewing of educational videos in children.

Although both the left and right IPS showed a strong relationship between neural maturity and children's math test scores, the right IPS showed an overall higher neural maturity score in the natural viewing paradigm and a more mathematics-specific neural response profile. As mentioned earlier, this finding is consistent with prior reports of the development of numerical processing in the brain [10],[11]; see [12] for review. Young children show number-related activations that are often stronger in the right hemisphere. Some have argued that right hemisphere IPS activations reflect more fundamental, early-developing numerical functions such as the comparison of analog quantities whereas the left IPS represents formal symbolic numerical content [8],[10],[11],[12],[15]. For example, Piazza and colleagues [8] showed that in adults, the left IPS is more involved in processing precise symbolic representations of numerical values than the right IPS. The precision of symbolic numerical representations increases throughout childhood. These findings thus predict that the left IPS will show a more protracted developmental trajectory than the right IPS. Our study confirms that prediction and expands the evidence to include both the amplitude and temporal pattern of children's neural responses in the IPS. In addition, our data provide novel evidence of a relationship between children's formal, school-based mathematics abilities and their neural responses in the IPS to a naturalistic education stimulus.

A traditional fMRI numerical task confirmed that both the left and right IPS regions responded selectively during basic numerical judgments compared to judgments of other categories in children. The IPS regions that exhibited number-related responses during the traditional paradigm overlapped math-related activations from the natural viewing paradigm. The overlap between number-related IPS activations across these tasks is impressive given that the tasks were quite different: the natural viewing task involved watching *Sesame Street* whereas the traditional task was numerical matching. Despite the fact that the stimulation and demands of the two tasks are very different, both tasks elicited activation patterns in the IPS that were selectively related to numerical processing. However, despite the overlap between the natural viewing and traditional paradigms, the naturalistic and traditional fMRI measures differed in their ability to explain variability in children's math performance. In both hemispheres, the natural viewing timecourse correlation of children-to-adults, or “neural maturity,” was more closely related to children's math performance than the traditional neural measure of BOLD amplitude from numerical stimuli in the IPS. The implication is that the natural viewing paradigm is better suited for predicting children's mathematics development than the traditional paradigm.

There are several reasons that the naturalistic stimuli and the measure of “neural maturity” could be ideal for predicting children's math performance. One reason is that the naturalistic neural maturity measure might better account for the richness of the whole BOLD timecourse, such as small and large scale fluctuations in activity. Brain regions that selectively respond to preferred information types could show subtle variation in the temporal response pattern to preferred information as well as to non-preferred information, e.g., [24]. Our finding of significant and sometimes math-specific intersubject correlations within both the numerical and non-numerical video content is consistent with that conclusion. That finding shows evidence of systematic neural responses to stimuli that elicit both high- and low-amplitude BOLD activity. Previous studies with adults [25] also have shown that natural viewing paradigms can reveal aspects of neural functioning that are not captured by the traditional measure of response amplitude, including variation in the temporal response windows of different brain regions and the sensitivity of different brain regions to temporal order in event sequences see [3] for review. Thus the naturalistic “neural maturity” measure has the potential to pick up a different set of neural response characteristics than the traditional measure of BOLD amplitude, particularly in the temporal dimension.

Another possible advantage of the natural viewing paradigm for studying children is that the natural viewing stimuli more fully engage the faculties that are used to learn in the real world. There is evidence that children's performance on reading, school readiness, and creativity tests improve after viewing educational programs such as *Sesame Street* [26]. Thus the content of educational videos, such as those used in the current study, can interact with children's school-based knowledge. The content of a real-world video might be a better stimulus for eliciting the suite of cognitive and neural processes that children likely recruit in school. These advantages of the natural viewing stimuli over a more traditional task with simple stimuli suggest that naturalistic studies of brain activity with real-world stimuli could serve as an important complement to highly controlled fMRI experiments on mathematics development.

We have reported a new set of analytic procedures for studying children's developing brain responses to complex real-world scenes. Complex real-world scenes simultaneously present multiple types of meaningful information across multiple modalities. The broad goal of this research is to understand how children's brains reflect signatures of the knowledge they have acquired, with the long-term goal of linking brain development to children's experiences and school performance [27]. We conclude that early in development, children exhibit dissociable, content-specific patterns of brain activity when left to view and think about educational material on their own. The degree to which children's brains elicit adult-like temporal patterns in their neural timecourses during natural viewing is a statistical predictor of their formal, real-world academic performance. Our data indicate that these complex stimuli can be used to identify individual differences in the brain mechanisms underlying children's real-world knowledge. The use of complex, real-world neuroimaging paradigms has the potential to advance our understanding of brain development in its natural context.

## Materials and Methods

### Participants

Twenty-seven typically developing children (ages 4.3 to 10.8 y, mean age = 7.1 y, SD = 1.6, 16 female) and 20 adults (ages 18.9 to 25.4 y, mean age = 20.7 y, SD = 1.7, 13 female) successfully participated in one or more of the experimental conditions (26 children and 20 adults in the natural viewing fMRI paradigm, 23 children and 20 adults in the traditional fMRI paradigm, and 19 children in the behavioral standardized testing). Children were excluded from conditions due to excessive head motion (>5 mm), opting-out, or experimenter error. The mean motion deviations for the remaining children (after online motion correction) were 0.39 mm translation (σ = 0.37) and 0.36 degrees rotation (σ = 0.24) in the natural viewing paradigm and 1.26 mm translation (σ = 1.33) and 1.5 degrees rotation (σ = 1.38) in the traditional paradigm. There was significantly less child motion in the natural viewing paradigm compared to the traditional paradigm (translation: *t*(21) = 3.5, *p*<0.005; rotation: *t*(21) = 3.97, *p*<0.005). The difference in child head motion between tasks is noteworthy considering that the natural viewing task was almost twice as long as the traditional task. Anecdotally, children seemed calmer and more engaged by the natural viewing task than the traditional task and this observation is empirically supported by the motion data.

All participants were screened for neurological abnormalities. All procedures were approved by the Research Subjects Review Board.

### Stimuli, Task, and Procedure

Prior to the MR scanning session, children were given a 30-min training session in a mock scanner to practice the experimental task, and remaining motionless during scanning. In the actual MR scanner, headphones, foam padding, and medical tape were used to secure the children's heads. Adults received verbal instructions and a brief session of task practice.

During the MR scanning session, we measured participants' neural activity (BOLD) during (1) a natural viewing paradigm and (2) a traditional fMRI paradigm with faces, shapes, numbers, and words.

#### Natural viewing fMRI paradigm

In the natural viewing paradigm, participants viewed a single 20.3-min montage of clips from children's educational television shows (Figure 1A). Individual clips ranged from 12 to 176 s in length and were edited into one continuous movie. The content of the video included letters, numbers, and other subjects (e.g., planets, shapes, Egypt). Participants were instructed to remain motionless while watching the movie but were given no instructions to fixate or restrict eye movement. A short quiz was administered at the end of the scanning session to ensure that participants attended to the movie.

#### Traditional fMRI paradigm

In the traditional fMRI paradigm, subjects compared pairs of stimuli presented on a computer monitor and reported whether the stimuli were the same or different. The stimuli consisted of pairs of isolated images (faces, numbers, words, or shapes) presented to the left and right of a central crosshair. Participants were instructed to fixate on the central crosshair throughout the scanning session. When a pair of stimuli was presented, participants were instructed to press a response button only if the two stimuli matched. No button press was taken to indicate a “non-match”. Fifty percent of the trials, distributed at random across each stimuli type, were “matches,” while the other 50% were “non-matches.”

In the “faces” condition, one face image was presented as a frontal shot; the other was presented as an oblique view. The faces were a “match” when both images were of the same person. On “numbers” trials, one image was an Arabic numeral between 1 and 9, and the other was a dot array. The images were a “match” when the number of dots in the dot array matched the Arabic numeral. The “words” condition consisted of trials in which two word images were presented, one in all capital letters in a serif font, and the other in all lowercase letters in a sans-serif font. The words were a “match” when both were the same word. On “shape” trials, two shape images were presented. A “match” occurred when both shapes were identical.

Stimuli were presented in 4.4-min runs in a blocked design. Each run consisted of three blocks per condition, with three picture comparison trials from the same condition per block. Each trial was presented for 2 s, followed by a 2-s inter-trial interval. 8 s of fixation followed each block. Blocks were semi-randomly presented.

Children were tested on two standardized IQ tests after the scanning session: the Test of Early Mathematics Ability, 3rd Edition TEMA-3 [28] and Kauffman Brief Intelligence Test, 2nd Edition KBIT-2 [29].

### MRI Parameters

Whole brain BOLD imaging was conducted on a 3-Tesla Siemens MAGNETOM Trio scanner with a 12-channel head coil at the Rochester Center for Brain Imaging. High-resolution structural T1 contrast images were acquired using a magnetization prepared rapid gradient echo (MP-RAGE) pulse sequence at the start of each session (TR = 2,530 ms, TE = 3.44 ms flip angle = 7 degrees, FOV = 256 mm, matrix = 256×256, 160 or 176 [depending on head size] 1×1×1 mm sagittal left-to-right slices).

An echo-planar imaging pulse sequence with online motion correction was used for T2* contrast (TR = 2000 ms, TE = 30 ms, flip angle = 90 degrees, FOV = 256 mm, matrix 64×64, 30 sagittal left-to-right slices, voxel size = 4×4×4 mm). The first six TRs of each run were discarded to allow for signal equilibration. The “movie” run of the natural viewing paradigm was one functional run of 610 volumes. The traditional fMRI paradigm was distributed over two to four functional runs of 132 volumes each. Total scanning time was approximately 40 min.

### fMRI Data Analysis

fMRI data were analyzed with the *BrainVoyager 2.1* software package and in-house scripts drawing on the BVQX toolbox in MATLAB. Preprocessing of the functional data included, in the following order, slice scan time correction (sinc interpolation), motion correction with respect to the first (remaining) volume in the run, and linear trend removal in the temporal domain (cutoff: two cycles within the run). Functional data were then registered (after contrast inversion of the first remaining volume) to high-resolution de-skulled anatomy on a participant-by-participant basis in native space. For each individual participant, echo-planar and anatomical volumes were transformed into standardized space [30]. Data from adults and children were normalized into the same Talairach space. The functional data from the traditional fMRI paradigm were not smoothed. A Gaussian spatial filter with an 8 mm full-width at half-maximum was applied to each volume for the natural viewing paradigm. We spatially smoothed the natural fMRI data because of the precedent set by Hasson and colleagues [2] for inter-subject correlations; we used the more conservative smoothing kernel (8 mm) of the two kernels tested in that prior study (8 mm and 12 mm).

Functional data from the traditional fMRI paradigm were analyzed using the general linear model (random effects analysis). Experimental events (duration = 10 s) in the traditional fMRI paradigm were convolved with a standard dual gamma hemodynamic response function. There were four regressors of interest (corresponding to the four stimulus types), one regressor for the button press, and six regressors of no interest, corresponding to the motion parameters obtained during preprocessing.

For the natural viewing fMRI paradigm, the data were pre-processed as described above for the traditional paradigm, and the resulting timecourses formed the basis for the intersubject correlation analyses. FD [31] was regressed out of each subject's timecourse to control for frame-to-frame head motion. FD is calculated by summing the absolute values of the derivatives from the six motion estimates of translation and rotation. Rotational displacements are converted to millimeters by projecting radians onto a sphere with a 50 mm radius (following Power et al. [31]). Subsequent analyses were performed on the residual timecourses after FD was regressed out. An additional control for signal intensity changes (DVARS following Power et al. [31]) is presented in Figure S2. That method removes volumes (1 back and 2 forward) surrounding timepoints where signal intensity changes by 0.5% or greater.

We implemented a developmental intersubject correlation method by correlating the timecourse of each voxel in the brain (for the whole 20-min video) for each child with the corresponding voxel in each adult (paired r-maps). In these children-to-adults correlations, we correlated each subject with every other subject (rather than correlating each child's data with an adult average) in order to be able to carry out parallel analyses for adults-to-adults and children-to-children without including the subjects' own data in the average. Additionally, this approach of correlating each subject with every other subject preserves the variability from individual subjects.

After obtaining paired r-maps for each child paired with each adult, we then calculated a mean image across the paired r-maps (a mean r-map) for each child. Each mean r-map represented that child's average similarity to adults. Each child thus had one r-map representing their mean similarity in neural activity to a group of adults at every voxel in the brain. These maps provide an index of how “adult-like” or, mature, each child's neural responses are across the brain, and are referred to as “neural maturity maps.”

We performed group-level statistics (one sample *t*-test on Fisher-transformed r values) over the children's “neural maturity maps” to plot the average group-level similarity of children's natural viewing BOLD timecourses to those of adults (whole brain). That analysis is shown in Figure 1. The same intersubject correlation method was used within-groups for the children-to-children and adults-to-adults correlation maps shown in the right two panels of Figure 1.

A whole brain analysis was conducted to measure the correlation between the children's chronological ages and their neural maturity maps (i.e., one correlation per voxel, between the vector of children's ages and their neural maturity values). Similarly, whole brain partial correlations between behavioral tests (TEMA, KBIT) and neural maturity were conducted over the children's neural maturity maps. The whole-brain partial correlations were conducted by regressing one test score out of the other and then correlating the residuals with neural maturity for each voxel, across the subject group. In addition, we also conducted ROI analyses. The regions tested in all ROI analyses were defined with independent data from their statistical tests. Note that some additional ROI data are shown from whole-brain analysis to illustrate individual subject scores. Percent signal change was calculated to illustrate the timecourses from the natural viewing paradigm. Percent signal change was calculated for each subject on the raw timecourse data by dividing each timepoint's intensity value by the mean intensity of the whole timecourse, then multiplying by 100 and subtracting 100. Statistical tests over response amplitudes from ROIs were conducted on the residual timecourses after the FD regression.

## Supporting Information

Figure S1 **Residual timecourses after correction for FD from the right IPS, left IPS, and Broca's area.** R values represent the overall timecourse correlation between the group of children and the group of adults. This figure is analogous to Figures 3 and 4 in the main article which show the raw timecourses from the same voxels for each region.
(TIF)

Figure S2 **The main result of our study is maintained when we apply the “scrubbing” method** [**31**] **to correct for signal intensity spikes in the timecourses.** This figure shows the dissociation in the whole brain correlation of neural maturity to math test scores versus neural maturity to verbal test scores after “scrubbing” has been applied. This figure is analogous to Figure 2, top panels, in the main article.
(TIF)

## Abbreviations

BOLD: blood oxygen level dependent
FD: framewise displacement
FDR: false discovery rate
fMRI: functional magnetic resonance imaging
IPS: intraparietal sulcus
ROI: region of interest
