# Supplementary figures and images for: Neural Activity during Natural Viewing of Sesame Street Statistically Predicts Test Scores in Early Childhood
Source: PLoS Biol. 2013 Jan 3;11(1):e1001462. doi: 10.1371/journal.pbio.1001462 (PMC3536813; doi:10.1371/journal.pbio.1001462)

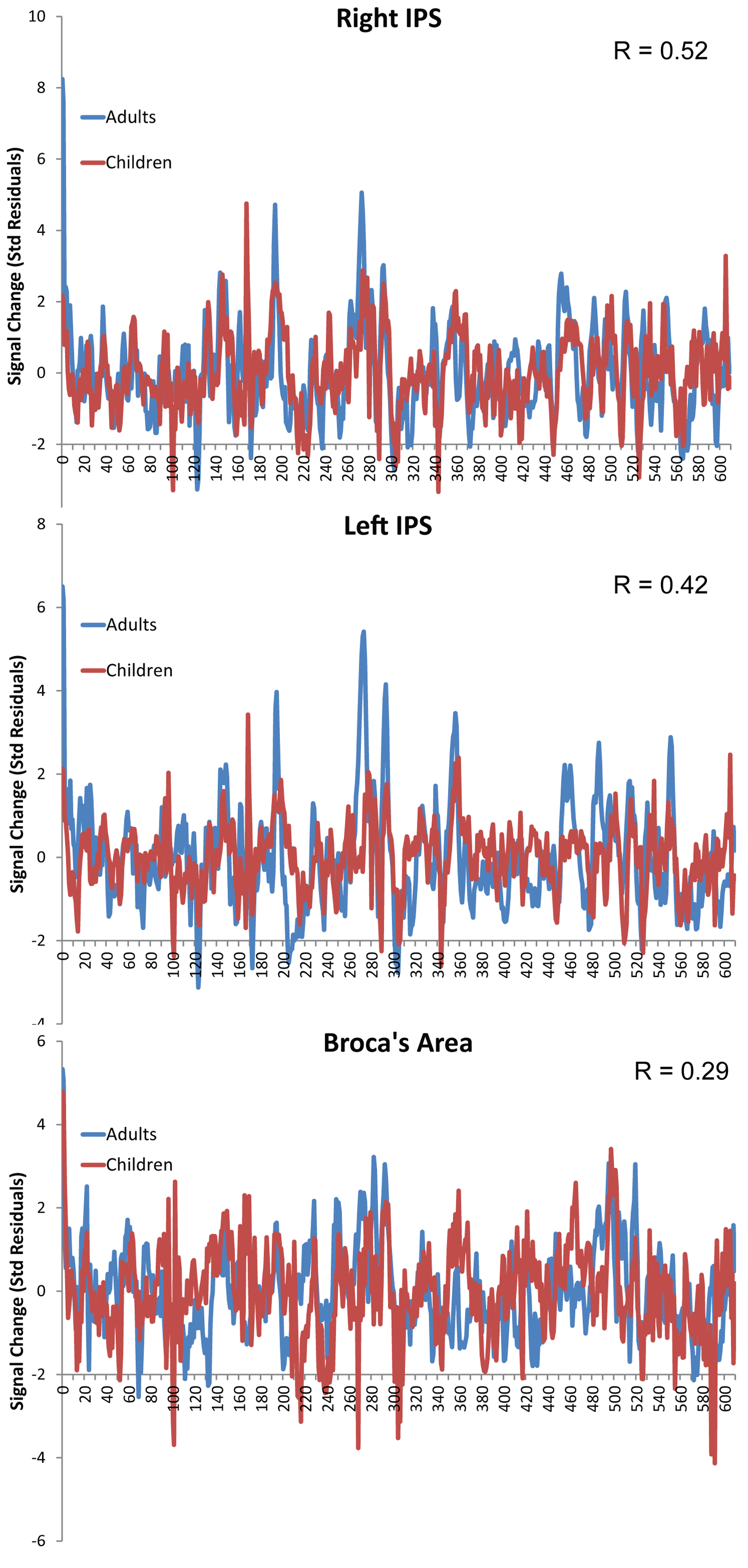

Supplement: Figure S1 — Residual timecourses after correction for FD from the right IPS, left IPS, and Broca's area. R values represent the overall timecourse correlation between the group of children and the group of adults. This figure is analogous to Figures 3 and 4 in the main article which show the raw timecourses from the same voxels for each region. (TIF) [file pbio.1001462.s001.tif]

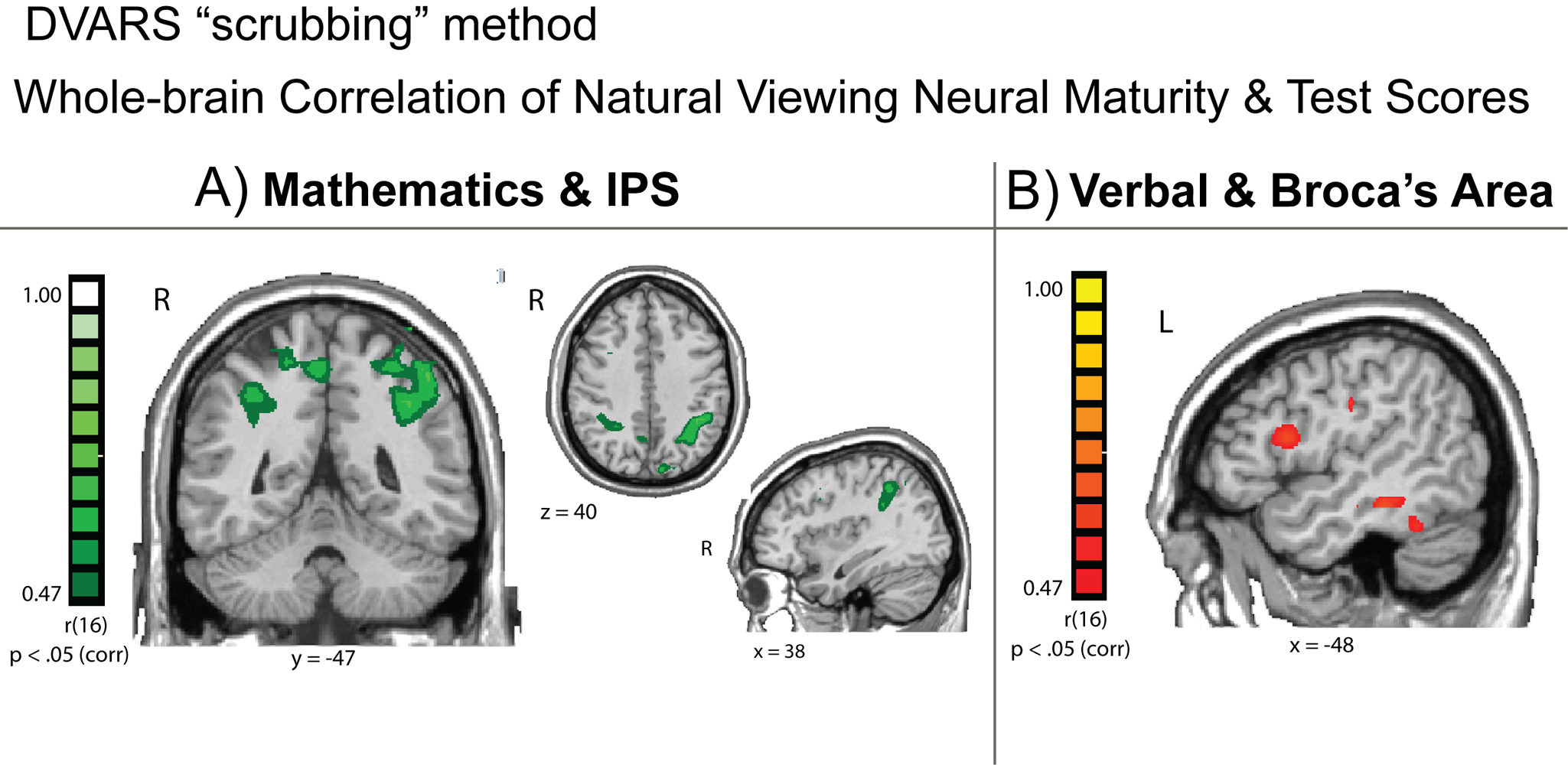

Supplement: Figure S2 — The main result of our study is maintained when we apply the “scrubbing” method [31] to correct for signal intensity spikes in the timecourses. This figure shows the dissociation in the whole brain correlation of neural maturity to math test scores versus neural maturity to verbal test scores after “scrubbing” has been applied. This figure is analogous to Figure 2, top panels, in the main article. (TIF) [file pbio.1001462.s002.tif]
